# Perceptions and attitudes of health science students relating to artificial intelligence (AI): A scoping review

**DOI:** 10.1002/hsr2.2289

**Published:** 2024-08-06

**Authors:** Shokoufeh Derakhshanian, Lucy Wood, Elio Arruzza

**Affiliations:** ^1^ UniSA Allied Health & Human Performance University of South Australia Adelaide South Australia Australia

**Keywords:** AI, artificial intelligence, education, health, healthcare

## Abstract

**Background and Aims:**

The recent integration of artificial intelligence (AI) across education, research, and clinical healthcare has led to a growing interest in AI training for healthcare students. This scoping review seeks to delve into existing literature, aiming to evaluate the perceptions and attitudes, of health science students toward the implementation of AI in their field.

**Methods:**

This review followed the methodological guidance offered by Arksey and O'Malley and the Preferred Reporting Items for Systematic Reviews and Meta‐Analysis extension for Scoping Reviews (PRISMA‐ScR). A systematic search was conducted in the databases Medline, Emcare, and Scopus. Studies using both quantitative and qualitative methodologies were eligible if they explored the perceptions or attitudes of health science students in relation to AI. Relevant data from eligible articles was extracted and analyzed using narrative synthesis.

**Results:**

Ten studies were included. Articles reported on the primary outcomes of perceptions (i.e., thoughts, ideas, satisfaction, etc.) and attitudes (i.e., beliefs, tendencies, etc.). Disciplines included nursing, diagnostic radiography, pharmacy, midwifery, occupational therapy, physiotherapy, and speech pathology were featured. Overall, students felt positively about the potential benefits AI would have on their future work. Students' interest and willingness to learn about AI was also favorable. Studies evaluating attitudes found positive correlations between attitudes toward AI, AI utilization, and intention to use AI. Negative perceptions related to threats of job security, and a lack of realism associated with AI software.

**Conclusion:**

Overall, evidence from this review indicates that health science students' worldwide hold positive perceptions toward AI. Educators should focus on instilling positive attitudes toward AI, given correlations between AI exposure and intention to adopt AI.

## 1 INTRODUCTION

Artificial intelligence (AI) is a dynamic field that involves the simulation of human intelligence through the use of computer science.^1^ The healthcare sector frequently encounters a range of challenges, including excessive workloads, inadequate funding, a shortage of skilled personnel, staff burnout, the challenges posed by an aging population, and unforeseen crises such as the COVID‐19 pandemic.^2^ Nevertheless, the incorporation of AI into healthcare offers the prospect of mitigating these challenges through various means.^2^ By leveraging advanced algorithms and data analysis, AI can enhance clinical decision‐making, optimize clinical workflows, allocate resources more effectively, alleviate workloads, and ultimately improve overall efficiency in healthcare settings.^3^ Further applications in healthcare, range from detection and diagnosis of disease, to analysis of treatments, predicting prognosis, and more personalized care.^4^

Globally, AI within the healthcare market is expected to grow to approximately US$67.4 billion by 2029, growing at a compound annual growth rate of 48% (Monash University, 2023). The recent integration of AI applications across education, research, and clinical healthcare has led to a growing interest in training healthcare students to be equipped with AI‐related knowledge and skills upon graduation.^3^ This recent surge signifies the recognition of AI's potential to revolutionize the healthcare industry by enhancing educational programs, driving innovative research endeavors, and transforming the way patient care is delivered.^3^

The role of current healthcare students, who represent the future stakeholders in healthcare, is pivotal in both the development and implementation of AI solutions within the healthcare industry.^2^ Extensive research involving medical students has demonstrated a range of perceptions and attitudes toward AI, ranging from optimism about the future to anxiety related to the possibility of being displaced by AI.^5^ However, limited consensus has been established as to whether similar notions are expressed by students involved in other health science disciplines. To the best of our knowledge, there exists no formal literature that has synthesized the current perspectives of students studying health disciplines which have potential AI implications, such as nursing, pharmacy, and diagnostic radiography. Without consensus, it may be difficult to tailor educational interventional interventions for health science fields specifically. Grunhut et al.,^6^ recently reported that most, if not all, medical schools in the United States of America did not incorporate AI in their curriculum. Several reasons, including a lack of faculty member expertise on AI, the often lengthy process for updating the curriculum, and deeply embedded values, may be hindering the inclusion of AI in the medical school curriculum. With limited research available on AI in health science curriculum, there is currently no widely agreed‐upon approach for introducing AI concepts to medical or health science students, nor is there consensus on what specific knowledge is most important.^7^ Hence, this scoping review seeks to “map out” the existing literature by synthesizing the current perceptions and attitudes of health science students toward AI within their field.

## 2 METHODS

The methodological framework described by Arksey and O'Malley was used for evaluating the extent of available evidence for this scoping review.^8^ This method involves the identification of relevant studies, study selection, charting the data, and collating, summarizing, and reporting the results. The independent screening and reviewing of eligible studies was consistent with the Preferred Reporting Items for Systematic Reviews and Meta‐Analysis extension for Scoping Reviews (PRISMA‐ScR).^9^

### 2.1 Eligibility criteria

This review encompassed a range of both experimental and quasi‐experimental study designs, including but not limited to randomized controlled trials, non‐randomized controlled trials, pre−post studies, and observational studies such as cross‐sectional studies, and case‐control studies. Clinical trials, previous meta‐analyses/reviews, editorial comments, and opinion pieces were excluded. Participants included undergraduate students within the discipline of health science. Studies that included primary, secondary, or postgraduate students were excluded, as well as practicing professionals. As there exists no recognized definition which specifically denotes disciplines under the “health science” banner, we chose to selectively include the fields that constitute the Australian National Registration and Accreditation Scheme,^10^ and comprise the overwhelming majority of health science and allied health clinical activity.^11^ Specifically, we included undergraduate students enrolled in nursing, pharmacy, physiotherapy, podiatry, medical radiation, optometry, speech pathology, midwifery, and occupational therapy.

Four inclusion criteria were used during screening. These included: (1) the study examined students' perceptions, attitudes, and/or knowledge of AI relating to their field of health science; (2) it presented empirical results; (3) it was published in a peer‐reviewed journal; and (4) it was written in English. Pertaining to criterion (1), articles were included if they featured any independent variable relating to perceptions (i.e., thoughts, ideas, satisfaction, etc.), attitudes (i.e., beliefs, tendencies, etc.), and knowledge (i.e., acquisition of theoretical concepts).

### 2.2 Search strategy

A systematic search was conducted in the databases Medline, Scopus, and Emcare to gather relevant literature published until January 2024. The search incorporated specific keywords including: [Deep learning or deep‐learning or AI or machine* intelligen* or comput* intelligen* or neural network* or machine learning] AND [higher education or tertiary education or university or college or further education or undergraduate or student] AND [allied health or health science or radi* or physio∗ or podiat* or occupational therap* or physical therap* or speech patho* or optometr* or nurs* or widwi* or pharm*] AND [knowledge or perspective* or perception* or skill* or thought* or opinion* or sentiment* or awareness]. The search was further refined to include only English language studies. Additionally, the reference lists of prior reviews and included studies were examined for additional relevant sources.

### 2.3 Data extraction

Two reviewers (E. S. A. and S. D.) independently extracted relevant data from the included studies. A data extraction table was created, which encompassed the following aspects: The characteristics of studies (publication year, sample size, country, discipline), participant characteristics (age and gender where possible), and characteristics relating to the educational intervention and outcome measures. Disagreements that arose between the reviewers were resolved through discussion, or a third reviewer (L. W.) if consensus was not achieved. When required, authors of studies were contacted to request missing or additional data.

## 3 RESULTS

**Figure 1:**
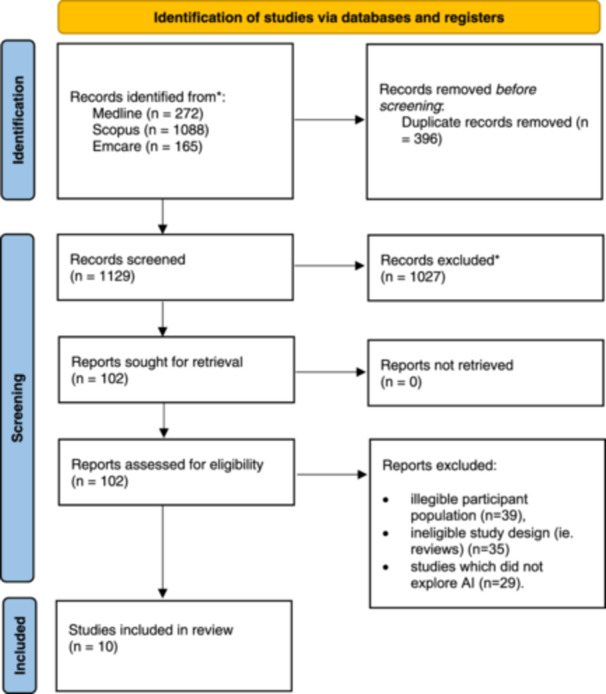
Adapted PRISMA (Preferred Reporting Items for Systematic Reviews and Meta‐Analyses) flowchart.

One thousand and five hundred twenty‐five studies were discovered, entailing 1088 from Scopus, 272 from Medline, and 165 from Emcare. Three hundred and ninety‐six duplicate articles were removed. Two reviewers (E. S. A. and L. W.) independently assessed the titles and abstracts of all studies found in the initial search. Discussions were undertaken to resolve discrepancies between reviewers. One hundred and two proceeded to full‐text screening. A total of 91 studies were deemed ineligible after full‐text analysis, due to ineligible participant population (i.e., students of non‐healthcare fields, or qualified health professionals) (*n* = 39), ineligible study design (i.e., reviews) (*n* = 35), or studies which did not explore AI (*n* = 29). Consequently, 10 studies were included in this review. Figure 1 presents a summary of this process in a PRISMA flowchart.

Table 1 summarizes the characteristics of all included studies. Publication dates of included studies spanned from 2021 to 2023. A global representation of studies was discovered, with inclusions from Saudi Arabia (*n* = 3), and single entries from South Korea, Singapore, China, Spain, Canada, Philippines, and Ghana. The majority of studies involved students from the nursing discipline (*n* = 7), whilst diagnostic radiography (*n* = 2), and pharmacy comprised the others. One multidisciplinary study sourced participants from midwifery, nursing, occupational therapy, pharmacy, physiotherapy, and speech pathology.^3^ The amalgamated total of participants across the studies was 3932, with individual sample sizes ranging from 22^12^ to 2167.^3^

**Table 1:** Characteristics of included studies.

Study	Year of publication	Field	Domain	Study design	Country	Number of participants	Sex (M/F)	Age	Years of study
Abdelaliem et al.	2022	Nursing	Quantitative	Cross‐sectional	SAU	697	0/697	<25^a^	2021
Ampofo et al.	2023	Diagnostic radiography	Quantitative	Cross‐sectional	GHA	181	97/82	21.7 (1.81)	NR
Chen et al.	2023	Nursing	Qualitative	Cross‐sectional	CHN	22	5/17	21−23^a^	2022
Kwak et al.	2022	Nursing	Quantitative	Cross‐sectional	KOR	210	27/183	23.55 (6.67)	2021
Labrague et al.	2023	Nursing	Quantitative	Cross‐sectional	PHL	200	43/157	20.215 (1.448)	2023
Liaw et al.	2023	Nursing	Mixed	Pre−post test	SGP	35	NR	NR	2021−2022
Qurashi et al.	2021	Diagnostic radiography	Quantitative	Cross‐sectional	SAU	46	138/86	NR	2021
Rodriguez‐Arrastia et al.	2022	Nursing	Qualitative	Posttest	ESP	114	23/91	22.71 (5.75)	2021
Syed and Al‐Rawi	2023	Pharmacy	Quantitative	Cross‐sectional	SAU	157	118/39	(18−30)^a^	2022−2023
Teng et al.	2022	Midwifery Nursing Occupational Therapy Pharmacy Physiotherapy Speech pathology	Mixed	Cross‐sectional	CAN	2167	805/1355	NR	NR

Abbreviations: CHN, China; ESP, Spain; GHA, Ghana; KOR, South Korea; NR, not reported; SAU, Saudi Arabia; SGP, Singapore.

a Range.

Eight studies employed a cross‐sectional study design. Three studies utilized an educational intervention relating to AI, before data collection occurred. Most studies were quantitative (*n* = 6), whilst three studies were purely qualitative, and two studies adopted a mixed methods approach. The outcome measures used varied, with studies using Likert scale questionnaires, focus groups, open‐ended questionnaires, and sometimes a combination of these measures.

### 3.1 Perceptions

Key findings of individual studies are summarized in Table 2. The term “perceptions” has been utilized to encompass any outcome based upon self‐assessment or self‐reflection as conducted by the students themselves; reported outcomes pertaining to this term included “perceptions,”^12^, ^13^, ^14^, ^15^, ^16^, ^17^, ^18^ “perspectives,”^3^ and “opinions.”^19^ Most studies reported that findings for these outcomes were overall of a positive nature.

**Table 2:** Key findings of included studies.

Study	Outcome	Outcome measure	Key findings
Abdelaliem et al.	Perceptions	Likert scale questionnaire	83.6% (583) of nursing students exhibited a high level of perception of AI. Overall, participants exhibited moderate AI readiness, high levels of technology acceptance, and high levels of AI technology adoption.
Ampofo et al.	Perceptions	Questionnaire	Three‐quarters of radiography students believed that AI would have an overall positive impact on medical imaging practice. Two‐thirds of participants felt threatened or unsure about their job security due to the incorporation of AI technology in medical imaging equipment. 90% of students were interested in learning more about AI and its use in medical imaging.
Chen et al.	Perceptions	Focus groups	Among nursing students, there was a limited understanding of the current state of AI. Concerns were raised about the potential loss of empathy if the chatbot design did not prioritize compassionate care. More than half of the participants preferred practicing with humanoid robots or cyborgs for a more realistic interaction, while some found virtual chatbots acceptable, flexible, and reproducible. Some students felt that chatbots might not provide an extremely realistic experience for history‐taking in the clinical setting, lacking in comprehending the emotional aspect of human interactions.
Kwak et al.	Attitudes	Likert scale questionnaire	This study aimed to identify the predictors of attitudes toward and intent to use AI‐based healthcare technologies among nursing students. Positive attitude toward AI predicted intent to use. Performance expectancy (the degree to which AI‐based healthcare technology is believed to be helpful) and self‐efficacy (confidence in using AI‐based healthcare technology) correlated with a positive attitude toward AI.
Labrague et al.	Attitudes, perceptions	Likert scale questionnaire	Student nurses had favorable perceptions of AI utilization in nursing practice, expressed high intentions to adopt AI technology, and held positive attitudes toward AI. Perceived AI utilization in nursing practice had a significant positive effect on student nurses' attitudes toward AI (*p* < 0.001) and their intention to adopt AI technology (*p* < 0.001).
Liaw et al.	Perceptions	Likert scale questionnaire & focus group	This study evaluated a virtual reality simulation (VRS) using an AI chatbot doctor. Nursing students had positive perceptions of AI‐enabled VRS, with high scores for the facilitation of learning and its ability to engage participants. Students indicated that the human‐like expressions and gestures of the AI doctor could be improved. Students saw the AI‐enabled simulation as a complement to face‐to‐face simulation, suggesting its use as a pre‐learning resource and the inclusion of a variety of clinical scenarios in the future.
Qurashi et al.	Perceptions	Likert scale questionnaire	Approximately half of the radiography students expressed concern about the potential threat to their jobs due to the implementation of AI (*p* = 0.038). Nearly all radiography participants displayed a keen interest in AI education and expressed their readiness to integrate it into their radiology clinical practice. Approximately 70% of students agreed or strongly agreed that AI is useful in clinical decision making, specifically in justifying examinations, and selecting imaging protocols based on clinical question and patient condition. Over 70% of students agreed or strongly agreed that AI will be useful in improving diagnosis, saving time, and will assist in personalizing imaging for patients.
Rodriguez‐Arrastia et al.	Perceptions	Questionnaire	This study assessed the acceptability and feasibility of using a chatbot for patient safety. Many participants (nursing students) found the chatbot helpful, providing availability, resolving doubts, and increasing confidence in handling complex situations with evidence‐based information. Participants had positive experiences with the chatbot, finding it brief, useful, and user‐friendly. Students expressed concerns about the unknown aspects of these innovations, data security, and privacy issues.
Syed and Al‐Rawi	Perceptions	Likert scale questionnaire	Approximately 70% of pharmacy students believed that AI is a tool that aids healthcare professionals and nearly 60% of students recognized that the widespread use of AI would help healthcare professionals improve. More than three‐quarters of students agreed that AI helps reduce errors in medical practice and believed that AI helps healthcare professionals to make accurate decisions. More than half agreed that AI‐related knowledge and skills should be incorporated into the academic curriculum. Overall, students agreed that AI‐related education is important for them, especially encompassing ethical challenges associated with AI applications.
Teng et al.	Attitudes	Likert scale & open‐ended questionnaire	Over half the students either lacked knowledge about AI or had an incorrect understanding of it. The majority believed that AI would impact their careers and anticipated its integration within the next 5 or 10 years. Students in midwifery displayed significantly less optimism regarding AI compared to other healthcare students. Physiotherapy students demonstrated higher levels of hopefulness toward AI compared to other healthcare students.

Abbreviation: AI, artificial intelligence.

A common pattern across the cohort of studies was the reporting of positive perceptions about the potential benefits AI would have on students' future work and patient care. Specifically, Syed and Basil discovered high rates of nursing students that believed that AI would aid healthcare professionals, would improve their work, reduce errors in medical practice, and help them make accurate decisions.^19^ This was similarly observed in radiography participants expressing AI's usefulness in clinical decision‐making, specifically in justifying examinations, selecting protocols based on clinical questions and patient conditions, improving diagnosis, saving time, and assisting in personalizing care for patients.^17^

Perceptions toward employment and job security were reported by radiography‐based studies. Ampofo et al. found that 65% of students felt threatened or unsure about their job security due to the incorporation of AI technology in medical imaging equipment.^13^ Similar findings were reported in another sample of radiography students, which found approximately half of the radiography students expressed concern about the potential threat to their jobs due to the implementation of AI.^17^

A common finding of favorable student interest and willingness to learn about AI was discovered. More than 90% of radiography students in both radiography‐based studies were interested in learning more about AI and its use in medical imaging and expressed their readiness to integrate it into their clinical practice.^13^, ^17^ Pharmacy students were somewhat less enthusiastic, with 56.7% of pharmacy students agreed that AI‐related knowledge and skills should be incorporated into the academic curriculum.^19^

Unique to nursing‐based studies, authors evaluated student perceptions of AI chatbots specifically, and some inconsistencies in perceptions are apparent. Rodrigues‐Arrastia et al. found nursing students had positive perceptions of the chatbot utilized in their study, finding it helpful in providing availability, resolving doubts, and increasing confidence in the students.^18^ Conversely, some students cited that chatbots might not provide an extremely realistic experience for history‐taking in the clinical setting, and lack in comprehending the emotional aspect of human interactions.^12^ Concerns about a lack of realism and patient empathy, was also a pattern found across nursing studies; participants preferred practicing with humanoid robots^12^ and yearned for human‐like expressions and gestures to be improved.^16^

### 3.2 Attitudes

Three studies explicitly explored “attitudes” toward AI. In the study by Labrague, findings suggest that student nurses hold positive attitudes toward AI, and express high intentions to adopt AI technology. Perceived AI utilization in nursing practice had a significant impact on their intention to adopt AI technology (*β* = 0.458, *p* < 0.001). This finding was supported in another nursing study, which found a positive correlation between attitude and intent to use AI.^20^

Teng's study was the only included entry which spanned across multiple health disciplines. Authors found those who were less advanced in their training had less favorable outlooks toward AI than upper‐year students who had greater knowledge of clinical practice (*p* < 0.001).^3^ They also found students in midwifery displayed significantly less optimism compared to other healthcare students, whilst physiotherapy students demonstrated higher levels of hopefulness toward AI compared to other healthcare students.^3^ Regardless of their specific healthcare program, students recognized the importance of acquiring basic AI literacy.^3^

## 4 DISCUSSION

To the best of our knowledge, this is the first scoping review to synthesize the evidence relating to the perceptions and attitudes of AI held by students from health science disciplines. We found 10 studies which captured student perspectives of AI within their disciplines. The recency of studies included in this review reflect the contemporary nature of the topic. Overall, evidence from this review indicates that overall, health science students' globally, hold positive perceptions toward AI.

A comprehension of the implications of AI is imperative for future practitioners. It is widely accepted that AI will change the clinical, scientific, economic, and ethical future of healthcare.^21^ Given the next generation of healthcare professionals will be entering an industry significantly more AI‐developed than when their training commenced, it is envisioned the findings from this review can help facilitate the successful implementation of future AI education. This review has also provided evidence that there is a desire for more AI knowledge to be incorporated in higher education from varying healthcare students, as some are unaware of aspects of AI technology.^18^

Job security and threats to career development posed by AI were explored across the cohort of studies. This was especially evident in radiography studies, potentially due to the recent development of digital radiography, and the radiographer's dependence on digital equipment in their role.^22^ Although nonmedical imaging studies did not report upon employment‐related perceptions, a recent Qatari study of 193 medicine, dentistry and allied health students found 40% expressed concern about a threat to job security from AI.^2^ It is evident that a significant proportion of students feel concerned about their employment prospects. This may spawn from apprehension that AI may diminish the demand for traditional healthcare professionals, or apprehension pertaining to the level of proficiency needed to work alongside AI effectively, not to mentions fears about the ethical implications associated with relying on it for aspects patient care. These uncertainties may lead students to question the permanence and longevity of future roles. This is despite positive predictions provided by the recent NHS Topol Review which maintains AI would augment rather than replace healthcare staff, allowing clinicians more time to prioritize patient care.^23^ This is supported by a 2021 report which predicted that the risk of job displacement in healthcare from “AI and related technologies” would be lower than that in other sectors, and experience the largest net employment increases of any sector over the next 20 years.^24^

Another intriguing finding entailed how exposure to AI during education correlated with more favorable notions toward it. It seems that students' positive attitudes and intention to use AI become fostered with greater use of AI during undergraduate education. This was discovered with nursing students particularly. By exposing students to AI early on in curriculum, this may allow an advancement of confidence and enthusiasm required to adopt AI in their future studies and imminent careers.

Generally, students in a range of health disciplines have expressed their preparedness and receptiveness to applying AI in their future careers. An approach of instilling AI‐related perceptions, knowledge, and skills within the undergraduate education setting, aligns with supporting the ever‐changing landscape of modern healthcare, as well as the stances of national healthcare bodies. The Medical Radiation Practice Board of Australia have emphasized that in meeting future workforce demands, education providers must develop curricula which provide an understanding of clinical applications of AI, and fosters graduates that are proficient in safely integrating AI into modern practice.^25^ The Topol Review also recommends that educational institutions develop a digital education strategy to improve digital literacy at an undergraduate level.^23^

Undoubtedly, barriers to integrating AI in health education include limited resources for training, and resistance to change among education and healthcare professionals. However, the long‐term implications of negative or unjustified perceptions toward AI may influence adoption from healthcare professionals, and ultimately, effect patient outcomes. After all, AI accuracy AI adoption by healthcare professionals has been shown to have a positive and significant impact on patient outcomes.^26^ Patients are more likely to accept AI‐driven interventions if they believe these technologies will enhance the quality of care they receive. Healthcare professionals who follow evidence‐based practice and are therefore knowledgeable and aware of AI are able to dispel the concerns of their patients and achieve buy‐in from their patients.

Educators can implement practical steps to enhance AI knowledge and attitudes. Institutions should look to offer hands‐on training, providing students with opportunities to gain practical experience with AI tools and technologies and familiarizing students with AI algorithms, data analytics tools, and machine learning techniques commonly used in healthcare. Furthermore, a collaborative approach from educational and clinical stakeholders is required to ensure that AI‐related concepts applied by clinicians is reflected in the case studies and tutorials featured within undergraduate education. Using clinically relevant resources, educators can focus on instilling positive attitudes toward AI and combatting negative ones, given the correlations between AI exposure, and intention to adopt AI. The featuring of industry experts and guest speakers in the creation and delivering of AI content may also provide realism for students, and being exposed to firsthand accounts of AI applications and success stories can inspire and motivate students.

Interprofessional education is a key aspect of modern health discipline education and accreditation. This presents an opportunity for AI to be integrated into the curriculum, as collaboration between healthcare students and students from other disciplines, such as computer science and engineering, can provide valuable insights into how AI can be applied to solve complex healthcare challenges. Similarly, demonstrating examples of AI technologies being used in healthcare settings to improve patient care will showcase the tangible benefits of AI in healthcare practice. More broadly, advocating for continuing professional development through the advertisement of conferences, workshops, online courses, and professional networking opportunities, will help raise awareness about AI.

This scoping review naturally possesses some limitations. Firstly, the review included only studies published in English. There always exists the possibility that the systematic search did not acquire all relevant literature as sought by the search strategy. Furthermore, many institutions may be conducting interventions without reporting it, and many of the eligible health science disciplines were not represented in the final selection of articles. However, this may simply represent where in the healthcare system AI has already been implemented and where it is yet to be utilized, with nursing and radiography being the most reported. On a positive note, the included studies used a variety of outcome measures to report on students' perspectives and attitudes toward AI. As such, there was a range of depth of information provided between the focus groups, open‐ended questionnaires, and Likert‐scale questionnaires used. Lastly, given the review's intention of providing an overview of the topic, it did not formally critically appraise the evidence for methodological quality.

## 5 CONCLUSION

Health science students will be pivotal in the development and application of AI in healthcare. As both theoretical knowledge and practical application of AI is wide‐ranging across health disciplines, the perceptions and attitudes of students is widespread. Practically, educators should focus on providing evidence‐based examples of AI with hands‐on training and industry input, promoting continuous professional development, and leveraging interprofessional collaboration. We encourage future research in this field to address gaps in understanding the full scope of AI's impact on healthcare practice and education and analyze how critical factors, such as socioeconomic and cultural aspects, affect student perspectives. It is crucial for students to receive evidence‐based education so that their perceptions are appropriately informed and, hence, enable them to play a central role in enhancing future clinical practice. It is hoped this review, which provided insights into the notions of current students, can assist healthcare organizations and policymakers in informing their strategies for integrating AI technology in healthcare settings and help advocate for educational reforms that incorporate AI education.

## AUTHOR CONTRIBUTIONS

All authors contributed to the conception or design of the work, the acquisition, analysis, or interpretation of the data. All authors were involved in drafting and commenting on the paper and have approved the final version. All authors have read and approved the final version of the manuscript.

## CONFLICT OF INTEREST STATEMENT

The authors declare no conflict of interest.

## TRANSPARENCY STATEMENT

The lead author Elio Arruzza affirms that this manuscript is an honest, accurate, and transparent account of the study being reported; that no important aspects of the study have been omitted; and that any discrepancies from the study as planned (and, if relevant, registered) have been explained.

## Data Availability

Data sharing is not applicable to this article as no new data were created or analyzed in this study. Elio Arruzza had full access to all of the data in this study and takes complete responsibility for the integrity of the data and the accuracy of the data analysis.
